# The Annexin A1 Receptor FPR2 Regulates the Endosomal Export of Influenza Virus

**DOI:** 10.3390/ijms19051400

**Published:** 2018-05-08

**Authors:** Fryad Rahman, Mohammad Chebbo, Noémie Courtin, Aurelien Fotso Fotso, Marie-Christine Alessi, Béatrice Riteau

**Affiliations:** C2VN Aix Marseille Univ, INSERM, INRA, C2VN, 13005 Marseille, France; fryad.rahman@gmail.com (F.R.); mohamad.chebbo1994@gmail.com (M.C.); noemie.courtin@gmail.com (N.C.); aurelien.fotso-fotso@univ-amu.fr (A.F.F.); marie-christine.alessi@univ-amu.fr (M.-C.A.)

**Keywords:** influenza, FPR2, Annexin A1

## Abstract

The Formyl Peptide Receptor 2 (FPR2) is a novel promising target for the treatment of influenza. During viral infection, FPR2 is activated by annexinA1, which is present in the envelope of influenza viruses; this activation promotes virus replication. Here, we investigated whether blockage of FPR2 would affect the genome trafficking of influenza virus. We found that, upon infection and cell treatment with the specific FPR2 antagonist WRW4 or the anti-FPR2 monoclonal antibody, FN-1D6-AI, influenza viruses were blocked into endosomes. This effect was independent on the strain and was observed for H1N1 and H3N2 viruses. In addition, blocking FPR2signaling in alveolar lung A549 epithelial cells with the monoclonal anti-FPR2 antibody significantly inhibited virus replication. Altogether, these results show that FPR2signaling interferes with the endosomal trafficking of influenza viruses and provides, for the first time, the proof of concept that monoclonal antibodies directed against FPR2 inhibit virus replication. Antibodies-based therapeutics have emerged as attractive reagents in infectious diseases. Thus, this study suggests that the use of anti-FPR2 antibodies against influenza hold great promise for the future.

## 1. Introduction

Influenza is an emerging and reemerging disease which is of particular concern to public health. Influenza outbreaks are usually associated with mild symptoms, such as cough, fever, headache, sore throat, sneezing and nausea, but can also result in severe illness and mortality. Every year, 250,000–500,000 people die from influenza globally [1,2,3].

Influenza is caused by an infection with an influenza virus. Four types of influenza have been described, namely, influenza A, B, C, and D; however, only influenza A, B, and C can infect humans, with influenza A (IAV) and B viruses being the most virulent. The virus life cycle of IAV begins with the attachment of the virus to the target cell through interaction of the viral hemagglutinin (HA) to sialic acids of the host cell [4,5]. This binding mediates internalization of IAV through a clathrin- or caveolae-dependent pathway and internalization of the virus into an endosome [6]. Then, to pursue its life cycle, the virus genome needs to be released from the endosome [6]. This step requires the fusion of the viral and endosomal membranes and is mediated by cleaved HA whichis activated by the acid environment of the endosome. Subsequently, the viral ribonucleoproteins (vRNPs) are released into the cytoplasm and reach the nucleus through a process involving the importins pathways [7]. In the nucleus, replication proceeds, in which transcription and replication occur. Viral proteins are synthesized in the cytoplasm and can either reach the plasma membrane or enter the nucleus to form new progeny of vRNPs [8]. An active process then allows the export of the vRNPs into the cytoplasm, where they can interact with the cell cytoskeleton and travel to the apical surface of the cell [9]. The nascent viruses then bud from the plasma membrane and are released from the infected cell.

Accordingly, the virion is composed of an envelope that comes from the host cell, in which are enchased both viral and cellular proteins [10,11,12,13]. Annexins are the most enriched cellular proteins of the virions; among them, annexin A1 (ANXA1) contributes to the virulence of influenza [12]. During viral entry to the host cell, not only HA binds to sialic acids but also ANXA1 binds to the formyl peptide receptor 2, leading to extracellular signal-regulated kinase (ERK) activation and an increase in viral replication [12,14]. However, how FPR2 promotes virus replication through ERK activation is unknown.

ERK activation is an important pathway during viral replication. It acts at two important stages of the virus life cycle. First, early activation of ERK facilitates the release of the genome from the endosome to the cytoplasm by promoting the vacuolar H+-ATPase (V-ATPase) activity and acidification of the endosome [15]. At later stages, it also allows the export of the vRNPs complexes from the nucleus to the cytoplasm [16]. In this manuscript, we investigated which of these two pathways is blocked by FPR2-mediated ERK activation during IAV infection. Our results showed that, during IAV infection, ANXA1/FPR2 permits the release of vRNPs from the endosome to the cytoplasm, thereby promoting IAV replication. This report also suggests that targeting FPR2 with monoclonal antibodies hold a great promise for treatment of influenza in the future.

## 2. Results

### 2.1. Treatment of A549 Cells with WRW4 Blocks IAVTrafficking

**Figure 1:**
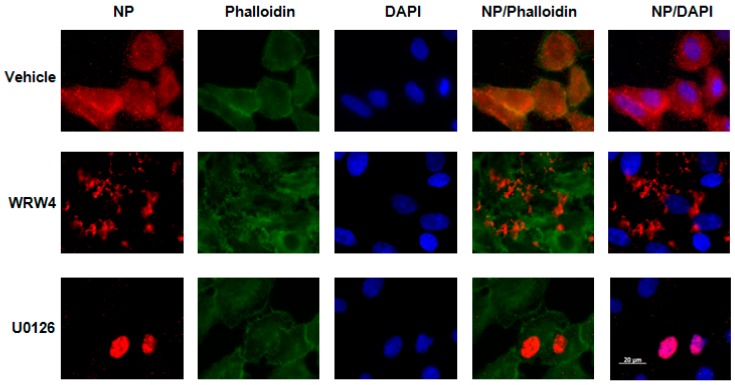
Localization of the NucleoProtein NP upon cell treatment with WRW4 and H3N2 infection A549 cells were infected with A/Udorn/72 virus (MOI 10) in the presence of vehicle, WRW4 (10 μM) or U0126 (50 μM). Four hours post infection, localization of the viral NP protein was assessed by immunofluorescence microscopy, using an anti-NP antibody. The actin cytoskeleton and nuclei were stained with phalloidin and DAPI, respectively. The merged images are shown. Scale bar, 20 µM.

**Figure 2:**
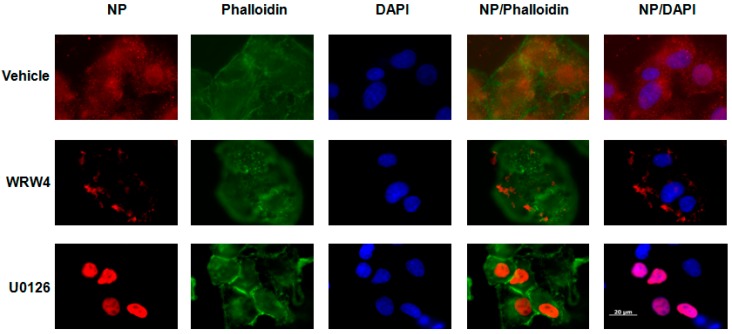
Localization of NP protein upon cell treatment with WRW4 and H1N1 infection A549 cells were infected with IAV A/PR/8/34 virus (MOI 10) in the presence of vehicle, WRW4 (10 μM) or U0126 (50 μM). Localization of the viral NP protein was assessed by immunofluorescence microscopy, using an anti-NP antibody, four hours post infection. The actin cytoskeleton (phalloidin) and nuclei (DAPI) were stained as well. The merged images are shown. Scale bar, 20 µM.

**Figure 3:**
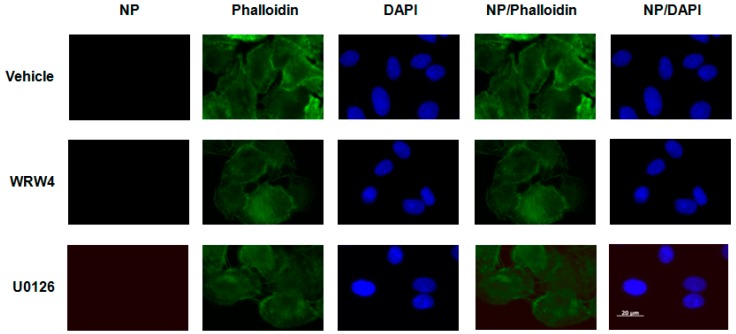
Specificity of the NP staining A549 cells were left uninfected and treated with either vehicle, WRW4 (10 μM) or U0126 (50 μM). Immunofluorescence microscopy was performed using an anti-NP antibody. The actin cytoskeleton (phalloidin) and nuclei (DAPI) were stained as well. The merged images are shown. Scale bar, 20 µM.

**Figure 4:**
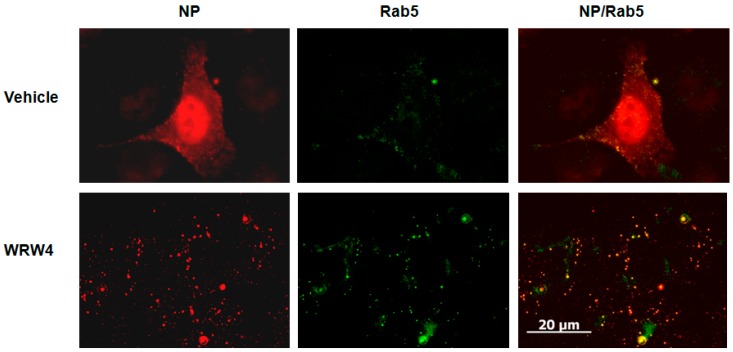
Colocalization of the NP protein with the Rab5 endosomal marker A549 cells were infected with IAV A/Udorn/72 virus (MOI 10) in the presence of vehicle or WRW4 (10 μM). Four hours post infection, localization of the viral NP protein was assessed by immunofluorescence microscopy, using an anti-NP antibody. The early endosome was stained using Rab5 antibody. Note that the Rab5 staining showed some background that was removed using ImageJ. The merged images are shown. Scale bar, 20 µM.

**Figure 5:**
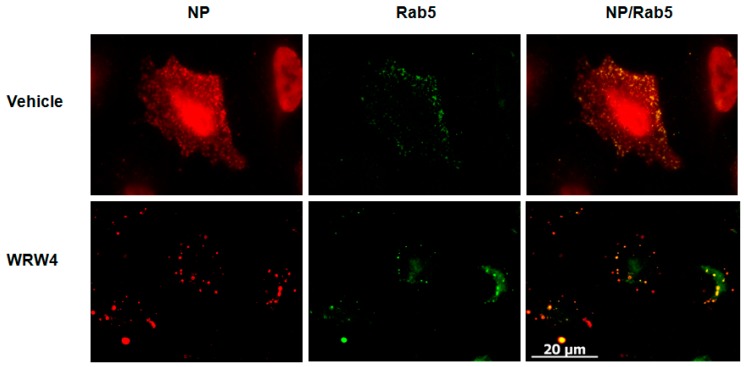
Colocalization of the NP protein with the Rab5 endosomal marker A549 cells were infected with IAV A/PR/8/34 virus (MOI 10) in the presence of vehicle, or WRW4 (10 μM). Four hours post infection, localization of the viral NP protein was assessed by immunofluorescence microscopy, using an anti-NP antibody. The early endosome was stained using Rab5 antibody. Note that the Rab5 staining showed some background that was removed using ImageJ. The merged images are shown. Scale bar, 20 µM.

To evaluate at which stage FPR2signaling modulates the IAV life cycle, we tested the effect of A549 cell treatment with the FPR2 antagonist WRW4 in the localization of the vRNPs complexes. This antagonist was chosen on the basis of its specificity towards FPR2 and not the other related FPRs [17].The viral nucleoprotein (NP) is a structural protein which encapsidates the virus genome [18], in which, following its expression, it is also the reflection of vRNPs trafficking. Thus, A549 cells were pre-treated with WRW4 (10 µM) or vehicle and infected with IAV A/Udorn/72 (H3N2) at a multiplicity of infection (MOI) of 10. As a control, cells were also treated with the ERK pathway inhibitor U0126. Four hours post infection, immunofluorescence staining was performed to assess the localization of the viral NP, using a specific anti-NP antibody. The nucleus and actin were also stained with DAPI and phalloidin, respectively. Results showed that, in untreated infected cells (vehicle), NP was largely distributed into the cytoplasm (Figure 1). However, upon cell treatment with the FPR2 antagonist, NP mainly accumulated into punctuated endosome-like vesicles. As previously described by Pleschka et al. [16], in the presence of U0126, NP was exclusively observed in the nucleus. This effect was independent on the virus strain as similar results were also observed upon infection with the A/PR/8/34 (H1N1) strain (Figure 2). In addition, no NP staining was observed in uninfected cells, showing the specificity of the NP detection (Figure 3). To confirm that endosome-like structures were indeed endosomes, immunofluorescence staining was performed using the endosomal tracker Rab5. A549 cells were infected with A/Udorn/72 (Figure 4) or A/PR/8/34 virus (Figure 5, MOI 10) in the presence of vehicle or WRW4 (10 μM). Four hours postinfection, localization of the viral NP protein was assessed by immunofluorescence microscopy, using an anti-NP antibody or an anti-Rab5 antibody; the merged images are also shown. Results showed that the staining of the NP protein colocalized with the one of the endosomal markers, Rab5. Altogether, these results indicate that blocking FPR2 led to an accumulation of NP in the endosomes and suggests that FPR2signaling interferes with the export of the vRNP complexes from the endosomes to the cytoplasm.

### 2.2. Treatment of A549 Cells with an Anti-FPR2 Antibody Blocks Virus Replication

**Figure 6:**
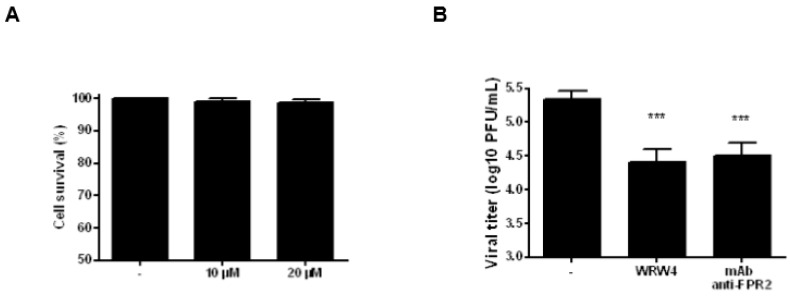
Cell viability and antiviral activity of the anti-FPR2 mAb (**A**) A549 cells were incubated with 10 or 20 µg/mL of the anti-FPR2 mAb for 24 h, and percentage of cell viability was assessed by trypan blue staining; (**B**) A549 cells were preincubated with 20 µg/mL of anti-FPR2 mAb or 5 µM of the FPR2 antagonist WRW4 and then infected with IAV A/PR/8/34 virus (MOI 1). 24 h after infection, infectious virus titers were determined by plaque assay. The Manne Whitney test was used for statistical analysis and results were considered statistically significant at *p* < 0.05 (*).

The potential offered by therapeutic-based antibodies has recently emerged [19]. Thus, we investigated the antiviral properties of the neutralizing anti-FPR2 mAb, FN-1D6-AI [20,21]. First, the cytotoxic effects of A549 cell treatment with 10 or 20 µM of the anti-FPR2 mAb was evaluated. Results showed no effect on cell cytotoxicity, by trypan blue staining (Figure 6A, 24 h post treatment). Then, A549 cells were pretreated with 10 µM of the anti-FPR2 mAb and infected with IAV A/PR/8/34 virus (MOI 1). 24 h post infection, infectious virus particles were evaluated in the supernatant by classical plaque assays. As shown in Figure 6B, A549 cells that were pretreated with the anti-FPR2 mAb released significantly fewer infectious viruses, compared with vehicle-treated infected cells. The effect of the specific anti-FPR2 antagonist, WRW4 (5 µM) was used as control. As expected, cell treatment with WRW4 significantly inhibited virus replication. Thus, these results showed that blocking FPR2 with the mAb, FN-1D6-AI significantly reduced viral replication in A549 cells.

### 2.3. Treatment of A549 Cells with the Anti-FPR2 mAb, FN-1D6-AI Affects Virus Trafficking

**Figure 7:**
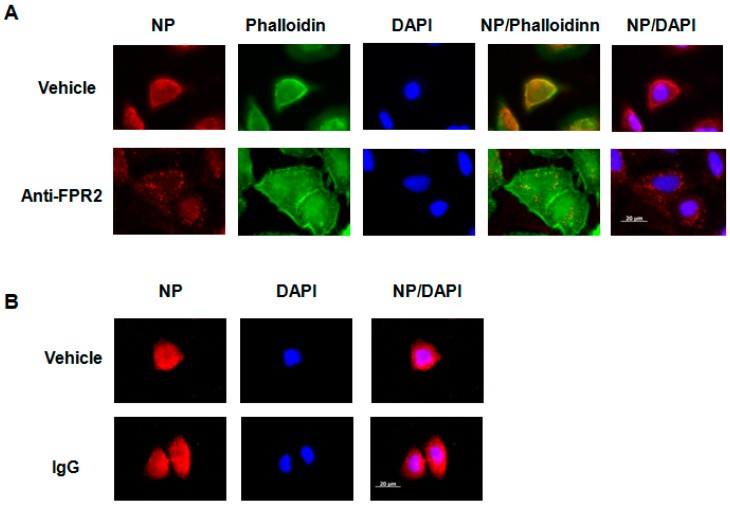
Localization of the NP protein upon cell treatment with an anti-FPR2 mAb A549 cells were infected with A/Udorn/72 (MOI 10) in the presence of (**A**) vehicle or the mAb anti-FPR2 (FN-1D6-AI, 20 µg/mL) or (**B**) vehicle or a monoclonal IgG control antibody (20 µg/mL). Four hours post infection, localization of the viral NP protein was assessed by immunofluorescence microscopy, using an anti-NP antibody. The actin cytoskeleton and nuclei were stained with phalloidin and DAPI, respectively. The merged images are shown. Scale bar, 20 µM.

**Figure 8:**
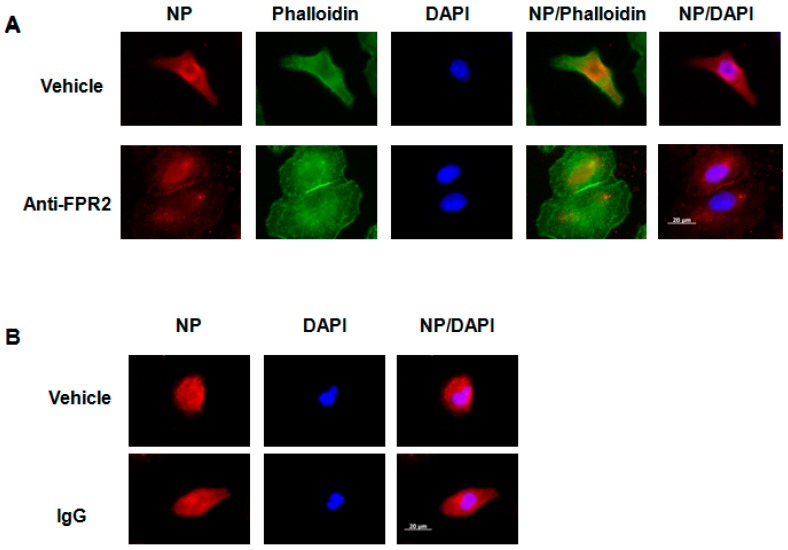
Localization of the NP protein upon cell treatment with an anti-FPR2 mAb A549 cells were infected with IAV A/PR/8/34 virus (MOI 10) in the presence of (**A**) vehicle or the mAb anti-FPR2 (FN1D6-AI, 20 µg/mL) or (**B**) vehicle or a monoclonal IgG control antibody (20 µg/mL). Four hours post infection, localization of the viral NP protein was assessed by immunofluorescence microscopy, using an anti-NP antibody. The actin cytoskeleton and nuclei were stained with phalloidin and DAPI, respectively. The merged images are shown. Scale bar, 20 µM.

To confirm the results obtained with the FPR2 antagonist WRW4, and to investigate whether cell treatment with the anti-FPR2, FN-1D6-AI would also affect virus trafficking in the endosome, A549 cells were pretreated with 20 µg/mL of the anti-FPR2 mAb and then infected with IAV A/Udorn/72 (H3N2, MOI 10). Four hours post infection, immunofluorescence staining of the viral NP was assessed. Results showed that, in contrast to untreated cells, where NP was broadly expressed in the cytoplasm, upon cell treatment with the anti-FPR2 mAb, NP was specifically observed in punctuated endosomes (Figure 7A). Cell treatment with an IgG control antibody had no effect on NP localization, showing the specificity of the anti-FPR2 antibody (Figure 7B). Staining of the nucleus (DAPI) and actin (Phalloidin) were also included as controls. Similar results were observed, although at a lesser extent, upon IAV infection with A/PR/8/34 virus (Figure 8A,B). Notably, although comparable virus release was found by plaque assay between WRW4 and anti-FPR2 pre-treatment followed by IAV infection, the punctuated endosome vesicles in cells treated with the antibody were not as clear as in cells treated with WRW4. This discrepancy was most likely related to technical issues and possible loss of efficacy of the antibody. Indeed, the viral plaque assay was performed using an unfrozen antibody, while a frozen antibody was used for immunofluorescence staining. However, because a difference was still observed, these results showed that blocking FPR2 with the mAb FN-1D6-AI affects virus trafficking in endosomes and subsequent virus replication.

## 3. Discussion

The present report supports an important role for FPR2 in the virus life cycle of IAV. Indeed, blocking FPR2signaling by cell treatment with a specific antagonist or a neutralizing antibody led to the accumulation of the viral NP proteins in the endosomes. Because NP is a structural protein that encapsidates the virus genome [18], it is reasonable to suggest that its localization is the reflection of vRNPs trafficking. Interestingly, our recent reports showed that FPR2 was exploited by IAV to increase its own replication through ERK activation [22]. ERK is a major pathway which promotes the V-ATPases-dependent acidification of the endosome, required for the fusion of the viral envelope with the endosomal membrane and subsequent release of the vRNPs into the cytoplasm. Taken together, our results suggest that FPR2signaling through ERK interferes in the early steps of the virus life cycle and enhances vRNPs release from the endosomes to the cytoplasm.

Interestingly, Arora et al. showed that, in A549 cells knock-down for annexinA1, the virus genome was preferentially present in the endosome and did not reach the nucleus as efficiently as in annexinA1-expressing cells [23]. During IAV infection, FPR2 was activated by AnnexinA1 that was incorporated into IAV particles [12]. Whether cellular annexinA1 can also activate FPR2 is not known; however, taken together, these results suggest that the effect observed by Arora and colleagues occured through FPR2 activation.

Annexins are proteins with multiple functions that bind negatively charged phospholipids in a calcium-dependent manner [24,25]. Annexins play important roles in many physiological and pathological contexts [26]. With regard to the modulation of IAV replication, not only annexinA1 but many other Annexins were involved as well. Annexin A2 permits the cleavage of plasminogen into plasmin-promoting HA cleavage and replication of low pathogenic viruses [10,27]. It also binds to the nonstructural 1 protein, increasing replication of highly pathogenic IAV [28]. Regarding AnnexinA5, it promotes IAV entry to the cell and blocks immune response to influenza, promoting virus replication [11,29]. In contrast to the proviral effects of annexinA1, A2, and A5, annexinA6 inhibits IAV replication, particularly by interfering with cholesterol homeostasis of late endosomes and virus budding [30,31]. Annexins do not only interfere with IAV replication but also with many other pathogens, as shown and reviewed recently [32,33,34]. Thus, a better understanding of the role of annexins in infectious diseases will help in the understanding of pathogens infections and subsequent development of novel therapeutics.

With regard to our results, they may be of relevance for developing future treatments against influenza, which still remains an important threat for public health. Unfortunately, the currently available drugs target viral proteins that have a high mutation rate [35]. Thus, the selection pressure in the presence of these drugs encourages the emergence of viral resistance; accordingly, the development of novel therapeutics against influenza is urgently needed [36]. Influenza is a parasite which requires cellular proteins to replicate. Thus, one promising strategy to limit the emergence of resistant viruses is to target a cellular factor required for efficient virus replication. For example, NF-kB or ERK are among the most critical factors promoting IAV replication [22,37,38,39]. Targeting such factors is very effective in experimental mouse models of IAV infection and also has shown successful improvement in the severity of the disease in infected patients in phase II clinical trials [40,41]. Interestingly, a FPR2 blockade also inhibitd ERK activation and was also very efficient in protecting against influenza in preclinical models of IAV infections [12,14]. It was demonstrated that not only did the use of FPR2 antagonists protect from IAV replication in the lungs but also inhibited harmful pulmonary inflammation during severe influenza [22]. Thus, the use of FPR2 antagonist is worth considering in future treatment of the disease. In addition, as has been shown here, the mechanism of action of FPR2 occurs through inhibition of the release of the vRNPs from the endosome. Because the current antiviral drugs against influenza, such as oseltamivir, act at a different level of the virus life cycle, i.e., through inhibition of the viral neuraminidase and virus release, this creates a path to the development of multi-drugs administration to treat the flu. Indeed, the combination of anti-FPR2 with oseltamivir has additive effects in the inhibition of virus replication [14]. In our study, we also found that a neutralizing anti-FPR2 mAb significantly inhibited virus replication in vitro. Antibodies-based therapeutics are emergentas attractive reagents in infectious diseases and beyond. In particular, monoclonal antibodies are considered to be more specific and potent compared with pharmacological reagents [19,42]. Accordingly, they are usually associated with fewer side effects in patients, compared with more conventional pharmacological tools. In addition, thanks to their large size, monoclonal antibodies have a longer half-life and slower clearance, compared withsmall molecules; thus, they can be highly advantageous in terms of pharmacokinetics. Thus, the proof of concept that cell treatment with a monoclonal antibody directed against FPR2 efficiently inhibits virus replication opens the door to novel anti-FPR2 immunotherapy against influenza.

## 4. Materials and Methods

### 4.1. Reagents Cells and Viruses

The following reagents were used in this study: antiviral M2 protein (Santa Cruz, Heidelberg, Germany), Alexa Fluor secondary antibodies (Life Technologies, Villebon-sur-Yvette, France), DAPI (4′,6′-diamidino-2-phenylindole, Sigma-Aldrich, Darmstadt, Germany), phalloidin (Invitrogen, Villebon-sur-Yvette, France), purple crystal staining (Sigma, Darmstadt, Germany), FPR2 antagonists WRW4 (Alomone Labs, Jerusalem, Israel), monoclonal anti-FPR2 antibody (clone FN-1D6-AI, Genova, Italy), monoclonal anti-Nucleoprotein (NP, HB-65, invivomab), conjugated Alexa Fluor 488 anti-Rab5 antibody (Santa Cruz, Heidelberg, Germany), IgG control antibody (ThermoFisher, Villebon-sur-Yvette, France), ERK inhibitor pathway U0126 (Sigma-Aldrich, Darmstadt, Germany). The human alveolar A549 and Madin-Darby canine kidney (MDCK) were a gift from G.F. Rimmelzwaan (Erasmus University, Rotterdam, The Netherlands) and were cultured as previously described [11]. IAV A/PR/8/34 (H1N1) was a gift from G.F. Rimmelzwaan (Erasmus University, Rotterdam, The Netherlands) and A/Udorn/72 (H3N2) was a gift from N. Naffakh (Pasteur Institute, Paris, France).

### 4.2. Infection Experiments and Cell Viability

A549 cells were preincubated with the FPR2 antagonist WRW4 or anti-FPR2 mAb (namely inhibitor) for 20 min before being infected with IAV A/PR/8/34 virus (MOI 1). After one hour adsorbtion, virus was removed and medium, which contained the inhibitor, was added. 24 h post infection, infectious virus titers were assessed in the supernatant by plaque assays. Cell viability in the presence of mAb anti-FPR2 was assessed by trypan blue staining, 24 h post treatment.

### 4.3. Titration Experiment

MDCK cells were seeded in a p6 culture plate (1.10^6^ cells per well) in DMEM 10% Foetal calf serum (FCS). The next day, serial dilution to the tenth of the samples was performed (diluted samples). After washing the cells twice with phosphate buffer saline (PBS), diluted samples were added for infection. After one hour adsorbtion at 37 °C for one hour, the supernatant was removed and MEM medium 2% agarose supplemented with1µg/mL of trypsin was added. After 72 h, agarose was removed and living cells were coloured with purple crystal for 5 min to detect plaque lysis. Cells were then washed extensively and infectious viruses were evaluated in each sample, with each plaque corresponding to one infectious virus.

### 4.4. Fluorescence Microscopy Experiments

A549 cells were seeded and cultured on glass coverslips in a multiwell plate. The next day, cells were preincubated with the specific FPR2 antagonist WRW4 (10 µM) or the anti-FPR2 antibody (20 µg/mL) for 20 min before IAV infection at a MOI of 10. After four hours post infection, cells were fixed with a 4% paraformaldehyde and permeabilized with 0.2% triton. Cells were then washed with PBS and incubated with primary antibodies to viral anti-NP, for 1 h at 37 °C. NP detection was performed using Alexa Fluor 594 secondary antibodies for 1 h at 37 °C. Endosomes were stained with a conjugated-Alexa Fluor 488 Rab5 antibody. Cells were counterstained with DAPI for 15 min and an Alexa Fluor 488 alpha-Phalloidin was added for 30min. Cells were washed with PBS and incubated with fluoromount medium. Images were taken by Zeiss IMAGER.M1. (Carl ZEISS, Oberkochen, Germany) and finally analysed using AxioVision Rel. 4.6 software.

## 5. Statistical Analysis

Virus titers statistical analyses were performed using GraphPad Prism software (version 5.0, GraphPad, La Jolla, CA, USA). The ManneWhitney test was used for statistical analysis and results were considered statistically significant at *p* < 0.05 (*).
